# Rosai–Dorfman disease differentiated from a posterior mediastinal tumour: a case report

**DOI:** 10.1093/jscr/rjae455

**Published:** 2024-07-11

**Authors:** Yoshimitsu Hirai, Yuichi Takahashi, Takahiro Kaki, Aya Shima, Kuninobu Kanai, Ryuta Iwamoto, Issei Hirai

**Affiliations:** Department of Cardiovascular and Thoracic Surgery, Wakayama Medical University, Wakayama, 641-8509, Japan; Division of Pathology, Naga Municipal Hospital, Wakayama, Kinokawa-City, 649-6414, Japan; Department of Respiratory Medicine, Naga Municipal Hospital, Wakayama, Kinokawa-City, 649-6414, Japan; Department of Breast and General Thoracic Surgery, Naga Municipal Hospital, Wakayama, Kinokawa-City, 649-6414, Japan; Department of Respiratory Medicine, Naga Municipal Hospital, Wakayama, Kinokawa-City, 649-6414, Japan; Department of Human Pathology, Wakayama Medical University, Wakayama, 641-8509, Japan; Department of Breast and General Thoracic Surgery, Naga Municipal Hospital, Wakayama, Kinokawa-City, 649-6414, Japan

**Keywords:** histiocytosis, mediastinal tumour, minimally invasive surgery, Rosai–Dorfman disease

## Abstract

Rosai–Dorfman disease presenting solely with intrathoracic lesions is exceptionally rare. Herein, we report the case of a 53-year-old man presenting with a posterior mediastinal tumour. Computed tomography revealed a 7-cm soft tissue shadow in the posterior mediastinum. Positron emission tomography-computed tomography demonstrated a high maximum standardized uptake value of 10.35 in the tumour, with no evidence of lymph node or other organ involvement. Serum marker levels were within the normal range. Thoracoscopic surgery was performed to obtain a biopsy for a definitive diagnosis and treatment planning. Postoperative histological findings revealed a diffuse infiltration of eosinophilic histiocytes, lymphocytes, and plasma cells. Immunohistochemical analysis indicated positivity for S-100 protein, oct-2, and cyclin D1 in these histiocytes. Consequently, the patient was diagnosed with Rosai–Dorfman disease and is currently asymptomatic, undergoing regular monitoring without treatment as an outpatient. The absence of characteristic findings, such as bilateral cervical lymphadenopathy, posed challenges in preoperative diagnosis.

## Introduction

Rosai–Dorfman disease (RDD) is a rare histiocytic disease of unknown aetiology characterized by painless bilateral cervical lymphadenopathy [1]. RDD has a prevalence of 1 in 200 000 [2], with only 2% of cases presenting solely with intrathoracic lesions [3]. In this report, we describe a rare case of RDD without bilateral cervical lymphadenopathy, which was differentiated from a posterior mediastinal tumour.

## Case report

**Figure 1:**
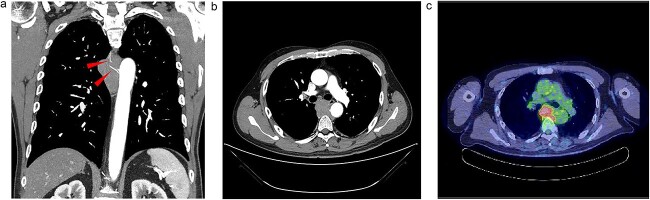
Radiologic findings. (a) A 15-cm soft tissue shadow in the posterior mediastinum of Th6-10, presenting as a multinodular mass. (b) Thoracic paravertebral soft tissue mass in the axial view. (c) Image of positron emission tomography-CT.

**Figure 2:**
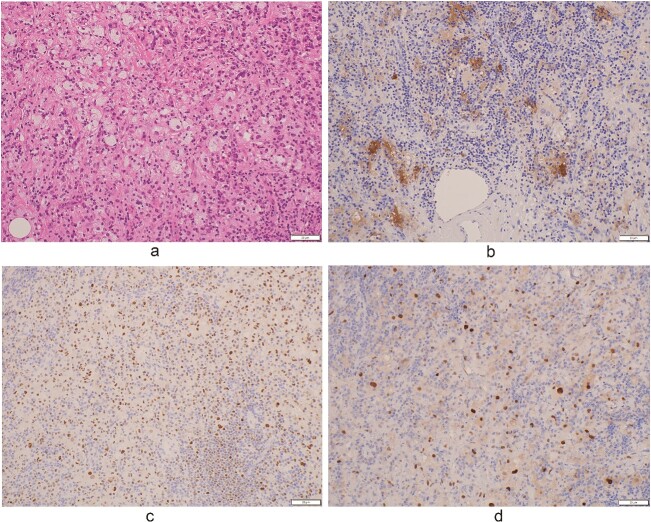
Histology. (a) Massive fibrosis with a storiform pattern, characterized by a cartwheel appearance of arranged fibroblasts and inflammatory cells [haematoxylin and eosin (HE) stain, ×10]. (b) Inflammatory cells, predominantly lymphocytes and plasma cells (HE stain, ×40). (c and d) Plasma cells staining positive for (c) IgG and (d) IgG4 immunohistochemistry (×40).

Upon examination for pneumonia during treatment for cerebellar infarction, a 53-year-old man was diagnosed with a posterior mediastinal tumour. He had a history of dyslipidaemia and had received appropriate medical treatment. Although the cerebellar infarction and pneumonia were in remission, the posterior mediastinal tumour persisted. He exhibited no systemic symptoms, and no abnormal physical findings were detected during a whole-body medical examination. Contrast-enhanced computed tomography (CT) revealed a 7-cm soft tissue shadow in the posterior mediastinum with clear and uniformly contrasted borders. There was no invasion into the adjacent vertebral body, and the blood vessels penetrating the interior were intact (Fig. 1a and b; red arrow). Positron emission tomography-CT (PET-CT) indicated a high maximum standardized uptake of 10.35 in the tumour, with no involvement of other lymph nodes or organs detected via whole-body imaging (Fig. 1c). Serum levels of various markers [including carcinoembryonic antigen, cytokeratin 19 fragments, beta-human chorionic gonadotropin, alpha-fetoprotein, soluble interleukin-2 receptor, immunoglobulin G, and serum C-reactive protein (CRP)] were within normal reference ranges. Endosonographic tissue confirmation via endobronchial ultrasound-guided transbronchial needle aspiration was inconclusive. Subsequently, thoracoscopic surgery was performed to obtain a biopsy for definitive diagnosis and treatment planning. The procedure, conducted using a complete thoracoscopic approach with three ports (7, 7, and 10 mm), revealed a solid hypertrophic lesion in the posterior mediastinum. A biopsy at two locations confirmed homogeneous, elastic, and soft tumours. The total operative time was 70 min with a bleeding volume of 30 ml. The patient experienced no postoperative complications and was discharged 7 days post-surgery. Postoperative histological analysis revealed a widespread collection of eosinophilic histiocytes, lymphocytes, and plasma cells (Fig. 2a). Immunohistochemical examination indicated positivity for S-100 protein, oct-2, and cyclin D1 (Fig. 2b–d), with an approximate IgG4-positive to IgG-positive plasma cell ratio of 24.7%. Consequently, the patient was diagnosed with RDD. The patient remains asymptomatic, is currently under outpatient monitoring without treatment, and showed no exacerbation of his condition up to 6 months post-surgery.

## Discussion

RDD, a non-neoplastic histiocyte proliferative disease, was initially reported by Rosai and Dorfman in 1969 [1]. This disease can manifest at any age, with a higher prevalence observed in males, typically at a mean age of 20.6 years [4]. However, recent cases have shown RDD diagnosis occurring at older ages than previously documented [5, 6]. The patient in this case report was 53 years old. Diagnosing RDD proved challenging as the patient presented with extranodal lesions, such as intrathoracic lesions, which are rarely associated with RDD and showed no evidence of bilateral cervical lymphadenopathy. PET-CT scans are valuable for assessing disease extent in patients with RDD, as recently reported [7]. They aid in identifying biopsy sites and evaluating treatment responses. However, the absence of bilateral cervical lymphadenopathy initially complicated the consideration of RDD as a potential differential diagnosis.

RDD diagnosis relies on histopathological examination, characterized by distinctive histiocyte proliferation (positive for S100 protein, oct-2, and cyclin D1) exhibiting emperipolesis (phagocytosed lymphocytes within histiocytes) [8]. While RDD is often perceived as a tumour and surgically resected, the prognosis is favourable for most patients, with spontaneous resolution observed in 20% of cases [9]. Treatment is recommended only for patients presenting with systemic symptoms or those with organ complications, such as central nervous system involvement [10]. Given the asymptomatic nature of the patient in this case, follow-up was recommended. The tumour was not invasive and had multiple blood vessels passing through it. We note that the lack of invasiveness and the intactness of multiple blood vessels presented herein may be characteristic of this condition.

In summary, we report a rare case of RDD in a patient solely characterized by extranodal lesions within the thoracic cavity. The absence of characteristic findings, such as bilateral cervical lymphadenopathy, posed challenges in preoperative diagnosis. These observations highlight the potential for RDD to manifest with atypical distributions of infrequent extranodal lesions, emphasizing the need for vigilant assessment to avoid delayed diagnosis and ensure favourable outcomes.
